# Evaluating tuberculosis knowledge and awareness of effective control practices among health care workers in primary- and secondary-level medical institutions in Beijing, China

**DOI:** 10.1186/s12879-024-09647-9

**Published:** 2024-08-02

**Authors:** Lijie Zhang, Xiaoge Ma, Menghan Liu, Sihui Wu, Zhili Li, Yuhong Liu

**Affiliations:** 1 Beijing Chest Hospital Affiliated to Capital Medical University, Beijing Tuberculosis & Thoracic Tumor Research Institute, Beijing, 101149 China; 2 Department of Epidemiology, School of Public Health, Cheeloo College of Medicine, Shandong University, Jinan, 250012 China

**Keywords:** Tuberculosis, Questionnaires, Health care worker, Comprehensive medical institutions

## Abstract

**Objective:**

Inadequate tuberculosis (TB) knowledge and awareness of proper TB control practices among health care workers (HCWs) may increase the risk of nosocomial TB transmission. This study aimed to assess HCWs’ TB-related knowledge and control practices to guide the development of more effective targeted TB health education and training programs.

**Methods:**

In January 2023 a cross-sectional survey was administered to 323 HCWs employed by five primary health care centers and three secondary comprehensive medical institutions in Beijing, China. Survey data were collected using a standard questionnaire.

**Results:**

Analysis of survey responses revealed TB knowledge and practices awareness rates of 60.4% and 90.6%, respectively. The overall average awareness rate across all 19 TB knowledge- and practice-related questions was 70.0%. Intermediate- and senior-level HCW’s average TB knowledge score was respectively 2.225 and 8.175 times higher than that of primary-level HCWs, while the average TB knowledge score of HCWs in secondary comprehensive medical institutions was 3.052 times higher than that of HCWs in primary health care centers. Higher average TB knowledge score correlated with higher-level professional titles and higher level work units, but higher average TB control practices score correlated with employment at primary health care center rather than secondary comprehensive medical institution. Notably, 13.6% of HCWs had not received TB training during the past three years, while 86.1% expressed willingness to undergo online TB training.

**Conclusion:**

These findings highlight inadequate TB knowledge and awareness of proper TB control practices among HCWs in primary health care centers and secondary comprehensive medical institutions in Beijing, underscoring the urgent need for targeted educational and training initiatives to improve TB awareness and control efforts.

Tuberculosis (TB) continues to pose a significant global public health challenge, partly because health care workers (HCWs), crucial for steming the tide of TB transmission, often fail to implement effective TB prevention and control measures. Notably, comprehensive medical institutions was the first point of identification for about 87.5% of the person affected by TB [1]. Furthermore, some previous studies showed a fact that lack of TB knowledge and awareness of effective TB control measures among HCWs [2–4], which contributed to diagnostic delays that support continued TB transmission, hampering TB control efforts. Although Beijing was one of the regions with the lowest TB epidemic in China, which the incidence rate was 22.7/100,000 in 2023, it is still a long way to achieve the goal of ending TB prosed by the World Health Organization (WHO). Currently, a large number of person in outpatient had respiratory symptoms in comprehensive medical institutions, and person affected by TB may mixed among them. If HCWs can identify person affected by TB in a timely and early stage, which will be conducive to further promoting early screening, early referral and reducing the nosocomial transmission of TB.

This study explored TB-related knowledge and control practices of HCWs in primary health care centers and secondary comprehensive medical institutions in Beijing. The findings of the study will be used to guide the development of more effective HCW-targeted TB education toward enhancing future TB control efforts.

## Methods

### Study setting

A cross-sectional survey was conducted in January 2023 across eight health institutions encompassing five primary health care centers and three secondary comprehensive medical institutions among Tongzhou, Yanqing and Changping districts in Beijing. This survey study was embedded within a prospective mass tuberculosis screening study among individuals with high risk factors of tuberculosis in three districts in Beijing, China, which supported by Beijing Science and Technology Major Project. The inclusion criteria for participating facilities included (1) one secondary comprehensive medical institution in each of three districts, (2) at least two primary health care centers in urban and rural areas respectively, (3) a large number of person seeking medical treatment, at least 50 persons average daily outpatient volume in last year, 4)Chest-X ray was available for TB screening. The total eight health institutions were selected including three secondary comprehensive medical institutions, two primary health care centers in urban area and three primary health care centers in rural area.

### Participants

The all HCWs worked at departments of general practice, respiratory, radiology, clinical laboratory and disease prevention in these eight health institutes were eligible for enrollment, including physicians, nurses, radiologist, microbiologists and public health officials. Workers not related with clinical and public health care were not included into this study. The total number of 335 eligible HCWs was obtained from managers/ directors of medical service section in all eight health institutions. The electronic survey link was distributed to eligible HCWs by managers/directors of medical service section. Eventually, a total of 323 eligible HCWs were investigated and enrolled in the study.

### Questionnaires and quality control

The survey questionnaire was meticulously crafted by the researchers to ensure comprehensive coverage of essential topics. It encompassed demographic details of the survey participants, such as gender, age, education level, professional title, work unit level, job position, working years, and participation in training programs. To gauge TB knowledge, respondents were presented with 13 key questions pertaining to TB epidemic, transmission, identification of high-risk groups, screening procedures, referral and registration protocols, diagnosis and treatment modalities, risk factors for acquiring TB, and the responsibilities of comprehensive medical institutions. The TB knowledge questions were initial developed based on the Core Information of TB Prevention and Control released by National Health Commission of the People’s Republic of China in 2017. Similarly, practices related to TB behaviors were evaluated through six pertinent questions, covering aspects like the designation of TB hospitals, handling of the person with presumed TB, ventilation and UV sterilization requirements for waiting areas, and self-protection measures. The TB control practices questions were developed based on the problems and disadvantages that obtained from two cross-sectional studies of TB medical institutions situation and implementation of TB prevention and control carried out by clinical center on TB, China CDC. Additionally, two questions focused on TB training and preferred methods for acquiring TB-related knowledge. All these question were further reviewed by the experts from public health and clinical fields of TB and revised. The questionnaire was tested by 10 HCWs to assess the time necessary to fill out the entire questionnaire, ensure questions were relevant, and test the clarity of the questions.

Each correct response for single answer questions earned a score of one, while incorrect responses received a score of zero. Multiple-Answers Questions earned a score of one for all correct answers selected. Yes/No questions earned a score for Yes response selected. These scores were meticulously tallied.

The questionnaire was seamlessly integrated into QuestionStar, facilitating the generation of QR code links. These links could be conveniently scanned using WeChat, enabling participants to effortlessly complete and submit the questionnaire. Each participant was allowed to fill out the questionnaire anonymously, ensuring confidentiality and encouraging candid responses. Furthermore, submission of the questionnaire was contingent upon completion of all questions, ensuring data completeness and accuracy.

Upon conclusion of the data collection phase, the investigators meticulously reviewed the information collected, leveraging the backend of QuestionStar.

The study was approved by the Ethics Committee of the Beijing Chest Hospital affiliated to Capital Medical University (2018-BJXK-030). An introductory letter stating the study purpose and promising confidentiality was sent to participants. The informed consent were obtained from all participants before starting to answer the questions.

### Definitions

The total TB knowledge score was calculated by dividing the number of correct answers provided by all participants by the total number of questions, followed by conversion of the resulting value to a percentage. The awareness rate for each TB practice-related question was calculated by dividing the number of participants who correctly answered that question by the total number of participants who answered the question, followed by conversion of the resulting value to a percentage. Average awareness rates served as threshold values that were used to judge awareness levels as good or poor, with rates above the average rate indicating good awareness and rates below the average rate indicating poor awareness. Primary health institutions are the smallest administrative level medical institutions in China, which are main responsible for disease prevention and care, and health promotion including vaccination, infectious disease monitoring, health education. Their main responsibilities for TB prevention and care system are to screen and refer the person with presumed TB, carry out TB contacting tracing and screening of high risk person with presumed TB, and medication management and health education for person with TB. While secondary comprehensive medical institutions refer to the regional hospitals that meet the Chinese hospital grade two standards, which provide comprehensive medical and health services to multiple communities and undertake certain teaching and scientific research tasks. Their main responsibilities for TB prevention and care system are to report person with presumed TB to national infectious disease network and refer them to designated TB hospitals for diagnosis and treatment [5]. Professional title is a symbol that reflects the professional technical level, achievement level and work ability of professional and technical personnel. The level of professional titles is generally divided into three levels: senior, intermediate and primary. QuestionStar is currently the largest professional online platform in China for questionnaire surveys, online exams, evaluations, and voting, which has the characteristics of easy and fast use, and low-cost production. WeChat is a free application that provides instant messaging services for smart terminals, which can real time send the voice messages, videos, images, text and documents by communication operators and operating system platforms over the network.

### Statistical analysis

Survey data were compiled using Excel 2019 and analyzed with SPSS 19.0. Descriptive data were summarized and presented as frequencies and percentages. Chi-square tests were conducted to evaluate awareness levels of participants with varying characteristics. Additionally, a multivariate logistic regression model was employed to analyze factors influencing levels of TB-related knowledge among HCWs. Results with *P* < 0.05 were considered statistically significant.

## Results

### Demographic characteristics of participants

As indicated in Table 1, a total of 323 completed questionnaires were collected from study participants. Among them, 72.4% were female. Participants were predominantly 30–39 years old (43.6%), 57% were bachelor’s degree, 42.4% held primary-level titles, and 58.2% were employed by primary health care centers.

The majority of participants worked in clinical positions (75.2%) and had more than 10 years of experience in their respective roles (66.5%). Notably, 86.4% had received TB-related training within the past 3 years. Additionally, 86.1% expressed a preference for online training to acquire TB knowledge.

**Table 1:** Demographic characteristics of participants (*n* = 323)

demographic factors	Number (%)
Gender	
Male	89 (27.6)
Female	234 (72.4)
Age	
20–29	60 (18.6)
30–39	141 (43.6)
40–49	68 (21.1)
≥ 50	54 (16.7)
Education level	
Master’s degree or above	66 (20.4)
Bachelor’s degree	184 (57.0)
College or below	73 (22.6)
Professional title	
Primary level	137 (42.4)
Intermediate level	120 (37.2)
Senior level	66 (20.4)
Work unit level	
Primary health care centers	188 (58.2)
Secondary comprehensive medical institutions	135 (41.8)
Job position	
Clinical	243 (75.2)
Medical technology	23 (7.1)
Public health administration	57 (17.6)
Working years	
≤ 5	69 (21.4)
6–9	39 (12.1)
10–19	123 (38.1)
≥ 20	92 (28.4)
Participation in TB training programs within 3 years	
yes	279 (86.4)
no	44 (13.6)
Preference for the ways of TB knowledge	
Online training courses	278 (86.1)
Online media (websites, microblogs, WeChat, etc.)	192 (59.4)
Offline face-to-face training	175 (54.2)
Paper promotional materials (books, brochures, etc.)	142 (43.9)
Continuing education in professional institutions	32 (9.9)

### Awareness of TB knowledge and TB control practices

The overall average awareness rate obtained for responses to all 19 TB knowledge and control practices questions was 70.0%. The comprehensive awareness rate for the 13 TB knowledge questions was found to be 60.4%. Notably, awareness regarding TB protective measures (Q8) was the lowest, reported at 2.8%. This was followed by basic knowledge of multidrug-resistant TB (Q11) at 13.6%, and understanding of the importance of TB control measures in primary health institutions and secondary comprehensive medical institutions (Q12), which stood at 32.2%. Conversely, responses to certain questions indicated strong knowledge among participants. For instance, awareness of TB transmission routes (Q4) was notably high at 98.5%. Similarly, recognition of TB classification as an infectious disease in China (Q1) was robust, with a reported awareness rate of 92.9%. (Table 2)

**Table 2:** Number of right answers to questions among health care workers

13 knowledge questions	Number (%)	6 practices questions	Number (%)
Q1: What type of infectious disease classified for TB in China? Category A Category B (right answer) Category C Category B managed according to A	300 (92.9)	Q14: Do you know the name and location of TB designated medical institutions in your city? Yes (right answer) No	315 (97.5)
Q2: What causes pulmonary TB? Tuberculosis virus Mycobacterium tuberculosis (right answer) Lung fluke inheritance	288 (89.2)	Q15: What should you do for the person with presumed TB in your clinical work? Provide anti-TB treatment in this unit Provide Symptomatic treatment in this unit Referral to other higher-level comprehensive hospitals Referral to designated TB hospital (right answer) Tell person to seek medical treatment at other hospital on their own	272 (84.2)
Q3: What are the suspicious symptoms of TB? Long term cough and phlegm ≥ 1month Fever, fatigue, loss weight ≥ 1month Cough, phlegm ≥ 2 weeks, or bloody sputum or hemoptysis (right answer) Chest pain and difficulty breathing	136 (42.1)	Q16: Do you know the ventilation requirements of waiting area for respiratory diseases? Yes (right answer) No	304 (94.1)
Q4: How is TB transmitted? Respiratory tract (right answer) Digestive tract Blood Insect-borne	318 (98.5)	Q17: Do you know the UV sterilization requirements of waiting area for respiratory diseases? Yes (right answer) No	302 (93.5)
Q5: What about the Epidemic situation of TB in China? The incidence rate of TB has declined rapidly in recent years It has been basically eliminated currently One of the infectious diseases with highest incidence and mortality rates The epidemic situation in rural areas is higher than that in cities (right answer)	150 (46.4)	Q18: Do you require the person in outpatient to wear masks? Yes (right answer) No	319 (98.8)
Q6: What is the main diagnostic criteria of TB? Typical symptoms and signs Chest X-ray or chest CT Bacteriological test of sputum (right answer) Serum TB antibody test	252 (78.0)	Q19: Do you wear N95 masks as required? Yes (right answer) No	244 (75.5)
Q7: How long should medical institutions reported to network reporting of communicable diseases for the person with presumed TB? 24 h (right answer) 12 h 48 h 2 h	221 (68.4)		
Q8*: What we can do to protect us against TB? Start preventive treatment once exposed to person affected by TB (right answer) Wearing mask correctly (right answer) Vaccination with BCG (right answer) Pay attention to hand hygiene after work	9 (2.8)		
Q9*: Who are the high risk population of TB that need to be actively screened? People living with HIV (right answer) People with low immunity (right answer) Person with diabetes (right answer) Elderly people (right answer) Person with pneumoconiosis and silicosis (right answer)	216 (66.9)		
Q10*: What are the treatment criteria of TB? First choice is the surgical treatment The treatment period for sensitive TB is at least 6 months (right answer) Regular follow-up exanimations should be conducted during treatment period (right answer) 90% person with sensitive TB can be cure with standardized entire treatment (right answer)	228 (70.6)		
Q11*: Common knowledge of multidrug-resistant TB The main first-line anti-TB drugs are ineffective (right answer) 4–5 drugs need to be used in combination, which is expensive (right answer) The treatment period can be as long as 18–24 months and there are severe adverse drug reactions (right answer) 90% person with sensitive TB can be cure with the entire treatment	44 (13.6)		
Q12*: What are the responsibilities for TB in primary health institutions and secondary comprehensive medical institutions? Screen and refer person with presumed TB (right answer) Diagnosis and treatment for person affected by TB Report person with presumed TB to National reporting system (right answer) Provide health education for person affected by TB (right answer)	106 (32.2)		
Q13*: What are the hazards of delayed diagnosis of TB? Increase the risk of TB transmission with medical institutions (right answer) Increase the risk of TB transmission in families and communities (right answer) Lead to treatment delay and some unnecessary treatments (right answer) Increase medical cost for person (right answer)	269 (83.3)		

*refered to multiple-answers questions

### Awareness of proper TB control practices

The comprehensive awareness rate for the six questions pertaining to TB control practices was 90.6%. Notably, the average awareness rate related to responses to the question “Do you wear N95 masks as required” was lowest (Q19) at 75.5%, followed by the corresponding rate for responses to “What should you do for patients with suspected TB in your clinical work” (Q15) of 84.2%. (Table 2)

Factors associated with awareness levels of TB knowledge and proper TB control practices.

The awareness level of TB knowledge varied significantly across different demographic and professional factors, including age (*P* = 0.003), education level (*P* = 0.015), professional title (*P* < 0.001), unit nature (*P* < 0.001), job position (*P* = 0.001), and working years (*P* = 0.020). Notably, individuals aged 40–49, holding a master’s degree or above, with senior professional titles, employed in secondary comprehensive medical institutions, holding clinical positions, and having ≥ 20 years of work experience exhibited higher levels of awareness.(Table 3).

Similarly, the awareness level of TB practices showed variations based on education level (*P* = 0.009), professional title (*P* = 0.016), unit nature (*P* < 0.001), and working years (*P* = 0.026). Participants with a college degree or below, holding primary professional titles, employed in primary health institutions, and with 6–9 years of work experience demonstrated a good level of awareness. (Table 3)

Multivariate logistic regression analysis revealed that individuals with intermediate titles (aOR: 2.225; 95% CI: 1.108–4.468) and senior titles (aOR: 8.175; 95% CI: 2.552–26.186) were more likely to exhibit better awareness of TB knowledge compared to those with primary titles. Additionally, HCWs working in secondary comprehensive medical institutions showed significantly higher awareness levels of TB knowledge compared to those in primary health institutions (aOR: 3.052; 95% CI: 1.631–5.712), and HCWs for job position of medical technology exhibited lower awareness of TB knowledge compared to clinical (aOR: 0.340; 95% CI: 0.129–0.889). However, HCWs in secondary comprehensive medical institutions exhibited lower awareness of TB practices compared to their counterparts in primary health institutions (aOR: 0.545; 95% CI: 0.312–0.953). (Table 4)

**Table 3:** Comparison of the awareness level of TB knowledge and proper TB control practices

Demographic factors	TB knowledge awareness level		TB practices awareness level	
	Average score	Above average, *n* (%)	Average score	Above average, *n* (%)
Gender				
Male	69.8	59 (66.3)	100	54 (60.7)
Female	69.5	143 (61.1)	100	136 (58.1)
χ^2^		0.739		0.174
*P*		0.390		0.677
Age				
20–29		34 (56.6)		38 (63.3)
30–39	69.3	76 (53.9)	100	91 (64.5)
40–49	68.3	52 (76.5)	100	35 (51.5)
≥ 50	71.9	40 (74.1)	100	26 (48.1)
χ^2^		14.075		6.464
*P*		0.003		0.091
Education level				
Master’s degree or above	69.8	50 (75.8)	100	28 (42.2)
Bachelor’s degree	69.7	114 (62.0)	100	114 (62.0)
College or below	68.8	38 (52.1)	100	48 (65.8)
χ^2^		8.374		9.521
*P*		0.015		0.009
Professional title				
Primary level	69.2	65 (47.4)	100	89 (65.0)
Intermediate level	69.2	78 (65.0)	100	72 (60.0)
Senior level	70.4	59 (89.4)	100	29 (43.9)
χ^2^		33.950		8.237
*P*		<0.001		0.016
Work unit level				
Primary health care centers	69.1	95 (50.5)	100	126 (67.0)
Secondary comprehensive medical institutions	69.9	107 (79.3)	100	64 (47.4)
χ^2^		27.679		12.480
*P*		<0.001		<0.001
Job position				
Clinical	69.4	166 (68.3)	100	141 (58.0)
Medical technology	68.4	9 (39.1)	100	14 (60.9)
Public health	71.2	27 (47.4)	100	35 (61.4)
χ^2^		14.437		0.260
*P*		0.001		0.878
Working years				
≤ 5	70.7	36 (52.2)	100	40 (58.0)
6–9	68.3	24 (61.5)	100	29 (74.4)
10–19	69.2	73 (59.3)	100	77 (62.6)
≥ 20	69.8	69 (75.0)	100	44 (47.8)
χ^2^		9.813		9.225
*P*		0.020		0.026

**Table 4:** Multivariate logistic regression for factors associated with the awareness level of TB knowledge and proper TB control practices

Variables	TB knowledge				TB practices		
	aOR	95%CL	*P*		aOR	95%CL	*P*
Age							
20–29	1				1		
30–39	0.456	0.190–1.096	0.079		1.512	0.623–3.666	0.361
40–49	0.560	0.142–2.199	0.406		1.556	0.437–5.543	0.495
≥ 50	0.372	0.073–1.909	0.236		1.464	0.329–6.511	0.616
Education level							
Master’s degree or above	1				1		
Bachelor’s degree	0.875	0.389–1.966	0.746		2.043	1.044–3.997	0.037
College or below	1.078	0.397–2.928	0.883		2.077	0.846–5.096	0.111
Professional title							
Primary					1		
Intermediate	2.225	1.108–4.468	0.025		0.837	0.417–1.679	0.617
Senior	8.175	2.552–26.186	<0.001		0.742	0.284–1.936	0.542
Unit nature							
Primary health care centers	1				1		
Secondary comprehensive medical institution	3.052	1.631–5.712	<0.001		0.545	0.312–0.953	0.033
Job position							
Clinical	1				1		
Medical technology	0.340	0.129–0.889	0.030		0.830	0.328–2.101	0.694
Public health	0.485	0.400-1.544	0.786		0.692	0.354–1.353	0.282
Working years							
≤ 5	1				1		
6–9	1.869	0.741–4.714	0.185		1.682	0.648–4.365	0.285
10–19	1.207	0.457–3.186	0.704		0.986	0.379–2.565	0.978
≥ 20	1.376	0.313–6.050	0.673		0.595	0.155–2.277	0.448

## Discussion

TB, a chronic airborne infectious disease, demands vigilant management to eradicate potential sources of infection, yet a significant proportion of HCWs employed by comprehensive medical institutions fail to maintain adequate vigilance against TB. This failure may be linked to HCWs’ limited understanding of TB as a highly prevalent disease that is most effectively controlled through early detection, which prevents long-term diagnostic delays that heighten the risk of TB transmission in hospitals, communities, and public places.

The findings of this study revealed that HCWs in both primary health care centers and secondary comprehensive medical institutions exhibited a low comprehensive TB awareness score of 60.4%, consistent with previously reported results [2, 3], underscoring the urgent need for improvement. Specific areas of lowest HCW knowledge included TB reporting and referral, screening, and diagnosis and treatment, findings consistent with previously reported results [4, 6]. Several factors likely contributed to these findings, including lower education levels of HCWs in primary health care centers and secondary comprehensive medical institutions, limited involvement of comprehensive medical institutions in TB diagnosis and treatment, and insufficient TB education and training [7–9]. Otherwise, this study was conducted in January 2023 which was just post Covid-19 pandemic and HCWs were mainly focused on epidemic prevention and control in primary health care centers and secondary comprehensive medical institutions in Beijing, which directly caused them to received insufficient training and education on TB. Given that primary and secondary comprehensive medical institutions serve as the frontline for identification of person with presumed TB, it is imperative for HCWs in these settings to possess a solid grasp of basic TB control, diagnosis, and treatment knowledge to enhance early detection efforts. Furthermore, one study from Botswana showed that multiple factors that influenced HCWs’ low TB contacting tracing included their knowledge, attitudes and practices [10]. Another study also demonstrated that HCWs’ capability impacted on their ability to engage in prevention and management of MDR-TB and the training was urgent need for HCWs [11]. This study results also underscored the importance of implementing targeted training programs aimed at enhancing the understanding of TB among HCWs in primary and secondary comprehensive medical institutions.

An analysis of factors influencing TB knowledge levels revealed a positive correlation between professional level or work unit level and TB knowledge, as consistent with findings of previous studies conducted in Guizhou province, Gansu province and Shenzhen city in China [12–14]. However, we observed a discrepancy wherein the awareness rate of TB practices among HCWs in secondary comprehensive medical institutions was lower than those in primary health institutions. This results may be related to the different responsibilities of the two types of institutions in TB prevention and care. Additionally, it’s noteworthy that the awareness rate of TB practices (90.6%) significantly surpassed that of TB knowledge (60.4%). This disparity may stem from the relatively simpler nature of TB practices questions compared to TB knowledge questions. Moreover, content pertaining to institutional infection control, which HCWs received thorough training and implementation for following COVID-19 control measures, likely contributed to this difference.

Several studies have demonstrated the importance of training frequency, HCW attention level, and HCW leadership level in influencing HCW knowledge and awareness [15, 16]. In this study, it was found that 13.6% of HCWs had not received any TB-related training within the previous three years, likely contributing to the poor TB knowledge awareness rate. Additionally, HCWs expressed a preference for online TB-training courses, followed by online media and paper-based promotional materials. This finding underscores the effectiveness of online training in continuing education and highlights its significance in HCW capacity building initiatives [17, 18]. These findings suggest a need to reinforce TB knowledge training among HCWs in primary and secondary comprehensive medical institutions. Moreover, implementing diversified approaches for capacity building is imperative to conduct training activities effectively. These approaches should be tailored to the specific needs of HCWs at different levels and positions, ensuring that training initiatives are targeted and comprehensive to meet the diverse needs of HCWs.

The limitations of this study stem from its cross-sectional survey design that primarily captures the situations of surveyed individuals in a single point in time, making it challenging to assess temporal relationships between antecedents and consequences based solely on the collected data. Additionally, study findings may not be widely generalizable to the other regions as we used the institutions recruited through three districts in Beijing. At last, the multiple-answers questions in the questionnaire were difficult to earn the score than the single answer questions, and 3 practice questions were subjective as respondents were asked if they do or do not know. These may be the reasons that the average score of TB control practices questions higher than TB knowledge questions.

In conclusion, while HCWs in primary health care centers and secondary comprehensive medical institutions exhibited good awareness of TB practices, their TB knowledge-related awareness was poor, particularly among HCWs with primary titles and those working in primary health care institutions. Urgent action is needed to implement targeted training programs focused on enhancing TB knowledge and capabilities of HCWs in primary- and secondary-level medical institutions. This approach is crucial for improving early identification of the person affected by TB, reducing diagnosis delays, and mitigating TB transmission risks.

## Data Availability

The data that supports the findings in this study can be made available through contacting the corresponding author under reasonable request.
